# Do neighborhoods have boundaries? A novel empirical test for a historic question

**DOI:** 10.1371/journal.pone.0313282

**Published:** 2024-12-02

**Authors:** Karl Vachuska

**Affiliations:** Department of Sociology, University of Wisconsin-Madison, Madison, WI, United States of America; Imperial College London, UNITED KINGDOM OF GREAT BRITAIN AND NORTHERN IRELAND

## Abstract

Do neighborhoods have boundaries? Scholars have debated how neighborhoods should be operationalized for decades. While recent scholarship has de-emphasized boundaries, I argue that boundaries are focal to understanding what neighborhoods are and why they are so segregated. Relying on everyday mobility patterns data from a panel of 45 million nationally representative devices, I demonstrate that divisions between contiguous census block groups in terms of everyday mobility patterns align with divisions in race, educational attainment, occupation, and age. Employing a novel clustering procedure, I further demonstrate that sets of census block groups can be easily separated in terms of differences in mobility patterns, indicating that meaningful clusters and boundaries within cities do exist. Subsequent results indicate these clusters are uniquely segregated in terms of race, educational attainment, and age, highlighting how other spatial aggregations can underestimate true segregation. Additional results suggest that unique social processes divide these clusters from one another, as indicated by exceptional variation in both COVID-19 case incidence and criminal offense timing. While I do not believe these clusters represent objective “neighborhoods,” I do assert that they can serve as a useful geographical unit for social analyses. These clusters may also be useful for measuring segregation in mobility patterns as well as for studying mobility network resiliency.

## Introduction

How to define neighborhoods is one of the oldest questions in Sociology and Urban Studies. Early scholars conceptualized neighborhoods as distinctive ecological niches that direct a set of social pressures onto residents [1, 2]. They posited that neighborhoods have their own unique characteristics and social processes that shape the behaviors of residents. For example, Shaw and McKay’s seminal work [2] identified that neighborhood poverty was strongly correlated with juvenile delinquency. They subsequently argued that the social disorganization present in these socioeconomically disadvantaged neighborhoods was a central cause of juvenile delinquency. Ultimately, these early ecological perspectives on neighborhoods emphasized the role of social factors in shaping the opportunities and challenges that residents face and highlighted the importance of understanding complex social processes in studying the dynamics of neighborhoods and their residents fully.

In the later 20^th^ century, neighborhoods became a focal component of understanding urban stratification processes [3]. Most studies have operationalized neighborhoods in an overtly simple and convenient manner, primarily relying on units of analysis that the Census Bureau or other government bodies have semi-arbitrarily designated. These units, such as census tracts, have commonly been used because they are readily available and easily accessible, despite the fact that they may not accurately reflect the best or most meaningful way to delineate neighborhoods [4]. Census tract boundaries, for example, are only required to align with a visible boundary (like a road) or a non-visible political boundary, and are not necessarily required to be socially meaningful [5]. Furthermore, except in exceptional circumstances, the outer boundaries of census tracts cannot be changed, meaning the boundaries of census tracts are not necessarily responsive to changes in meaningful boundaries. In spite of these issues, the neighborhood effects literature has come to rely heavily on these administrative units to measure neighborhoods and subsequently investigate how neighborhood characteristics are associated with individual outcomes.

More recently, scholars have pushed to shift away from operationalizing neighborhoods as units with non-overlapping boundaries. Hipp and Boessen [6] specifically proposed using “egohoods” or egocentrically-defined neighborhoods that overlap like waves. Hipp and Boessen justify the operationalization based on four bodies of literature: the network literature on the spatial distribution of social ties, the daily activities pattern literature, the mental mapping literature, and the travel to crime literature. Hipp and Boessen argue that where one resides is the center of one’s world in many ways. Social ties, activity patterns, self-described neighborhood boundaries, and behavioral patterns all tend to operate as distance decay functions.

While arbitrarily defining neighborhood boundaries is unideal, so is defining egocentric neighborhoods with a constant fixed radius. An egocentric perspective wrongly assumes that distance is *all that matters*. Subsequently, an egocentric perspective asserts that there are no boundaries between neighborhoods but that neighborhoods are like fluid waves rolling across the city. Hipp and Boessen’s [6] egohoods consist of concentric circles surrounding a single smaller geographical area, with the intuition being that the outcomes of that small central area are best predicted in terms of the attributes of the space within a fixed distance. Without consideration of additional boundaries, such an approach implies that distance matters exactly the same everywhere, despite the fact that certain boundaries mean certain proximal spaces are less connected than others. While the egocentric approach may be useful in some individual-level cases, it is unhelpful in conceptualizing what neighborhoods are. Ethnographic work strongly indicates that certain boundaries do exist, whether somewhat fuzzy or somewhat abrupt. Relatively small physical structures, such as roads or railroad tracks, can constitute stark boundaries dividing different groups of people that experience widely disparate social and environmental conditions [7, 8]. Beyond conceptual accuracy, operationalizing neighborhoods with non-overlapping boundaries is statistically simpler and produces results that are easier to interpret.

Broadly, both past approaches are flawed as they fail to assess and represent demographic segregation accurately. For example, stark racial segregation between a pair of adjacent census block groups turns into an apparently integrated census tract upon aggregation. Similarly, the egocentric approach depicts individuals living near a racial segregation border as living in an integrated neighborhood despite likely associating more greatly with one part of the neighborhood than the other.

Emphasizing demographic boundaries between neighborhoods is crucial so far as those demographic boundaries reflect divisions in social processes, which they often do. While attempting to draw lines that align with racial segregation blindly is inaccurate, so is ignoring the boundaries altogether. These boundaries are, in fact, likely to be the most important separations that can exist between any neighborhoods, and understanding where they lie can be of huge benefit to our ability to contrast neighborhoods and understand division. Without emphasizing them, other artificial boundaries may suggest division where it does not exist and fail to identify segregation where it does exist. Recent sociological research highlights the importance of boundaries in understanding racial segregation and inter-racial dynamics [9].

Ultimately, a focal reason for operationally defining a neighborhood is to understand neighborhood social processes and effects. By studying the outcomes that occur in specific neighborhoods (e.g., violent crime) or the outcomes of people who live in specific neighborhoods (e.g., mortality), scholars can better understand how neighborhoods affect residents. Galster [10] put forth a theory of the mechanisms of neighborhood effects. Perhaps the largest of the four categories Galster offered was “Social-Interactive” mechanisms. These mechanisms pertain to the social processes that occur within neighborhoods. Galster also describes a set of environmental mechanisms, referring “to natural and human-made attributes of the local space that may affect directly the mental and/or physical health of residents without affecting their behaviors”. Geographical mechanisms are similarly defined as “aspects of spaces that may affect residents’ life courses yet do not arise within the neighborhood but rather purely because of the neighborhood’s location relative to larger-scale political and economic forces”. Lastly, Institutional mechanisms refer to “actions by those typically not residing in the given neighborhood who control important institutional resources located there and/or points of interface between neighborhood residents and vital markets”.

Public health scholars have similar theories regarding the geographical attributes that are relevant to understanding health disparities in particular. Diez Roux and Mair [11] write that the physical and social environment processes in a neighborhood interact to drive the health outcomes of a neighborhood. They further highlight the challenge that comes with conventional methods for neighborhood measurement, “For example, small areas may be relevant to processes involving social interactions between neighbors, whereas larger areas may be relevant for food shopping behaviors. In some cases, the spatial context relevant to a particular process and outcome may not be commonly thought of as a “neighborhood” by residents. In addition, spatial contexts other than residential such as work contexts may also be relevant”.

While most scholars agree that neighborhoods are socially constructed, it is still important to identify approaches for empirically representing geographical aggregations in order to study social processes [12]. While not referring to these units as “neighborhoods” explicitly, geographical units of analysis can, and should be, theoretically informed by the concept of neighborhoods. Identifying the appropriate boundaries of the relevant geographical units is challenging, however. I propose that, at a minimum, geographical units of analysis in the social sciences should have two qualities. First, they should be geographically tight and spatially cohesive. This aligns with past scholarly thought. Siordia and Wunneburger propose that one can measure structural phenomena with a geographical unit only if the units are: “made up of geographical polygons that are (2) non-overlapping, (3) contiguous, (4) non-porous, and (5) non-fluid.” Siordia and Wunnenburger particularly emphasize the principle of spatial contiguity, which they describe as “an implicit and necessary condition” [12, 13]. Siordia and Wunnenberger outline polygon fragmentation as posing five issues for spatial data analysis: “(1) fallible polygon centroids; (2) indeterminable inaccuracy of macro-level measures assigned to micro-units across fragments; (3) variation in precision for micro-units’ approximate physical location; (4) misleading aggregations; and (5) ambiguous areal interpolations from fragments.” In terms of understanding residential life, having geographical units consisting of a sole spatially cohesive component ensures that residents reside in approximately the same area and experience essentially the same residential context. This requirement particularly aligns with the motivation for Hipp and Boessen’s “egohood”. Like Tobler’s first law of geography, research on social activity patterns and mental mapping suggest that more activity occurs near where people live rather than far [14]. As a result, tight, cohesive neighborhoods most optimally align with these properties.

The second primary quality I propose for geographical units is that their residents also should tend to experience similar organizational and institutional contexts in everyday life. For example, a neighborhood’s children should attend similar schools, employed adults should work in a similar set of workplaces, and everyone should tend to do their retail shopping at a similar set of stores. Well-defined neighborhoods should, ideally, not span the boundaries of multiple school districts, as children will not have a uniform schooling and socialization exposure. Similarly, a neighborhood should not geographically span two distinguishable areas that employ distinct classes of workers or be residence to structurally distinguishable groups of people. This logic is in line with past research. Most neighborhood clustering algorithms attempt to minimize heterogeneity within units and maximize between units [6, 15]. This idea has also been extended to social relations, with some scholars positing that neighborhoods should be crafted to maximize social ties within the neighborhood and minimize them outside of the neighborhood [16].

I would emphasize that this notion does not imply that neighborhoods cannot be desegregated demographically. Rather, I would highlight that the presence of homogenous communities is generally a strong indicator that the geographical boundaries between these communities constitute a relevant structural division. Sampson and colleague’s [17] neighborhood clustering approach generated internally homogenous clusters of census tracts in terms of race, education, income, and occupation (in addition to housing density and family organization), highlighting these as “key census indicators” with which it is important for neighborhood clusters to be internally homogenous. I additionally consider age, which has been increasingly studied in terms of residential segregation [18]. Indeed, theory suggests that residential location and movement in space should be associated with patterns of segregation in terms of race, occupation, and age [19–21]. Legacies of systemic racism are likely to structure both movement patterns and residence differently for different racial groups [19]. Additionally, heterogeneity between “micro-classes” would suggest similar distinctions in terms of movement patterns and residence for different occupational groups [20]. Ultimately, identifying relevant structural divisions between homogenous communities is paramount to understanding geographical disparities and implementing policy to address them. In this sense, my conceptualization of a neighborhood emphasizes homogeneity within communities because it offers an effective method for partitioning geographical regions into relevantly distinctive components and for understanding inequality broadly.

Ultimately, Galster’s framing of neighborhood effects provides straightforward justification for the two aforementioned qualities—that neighborhoods should be spatially cohesive, and residents should experience similar organizational and institutional contexts in everyday life. First off, the proximal attributes of a neighborhood affect the neighborhood. Environmental mechanisms like decayed physical conditions or toxic waste exemplify this. By operationalizing neighborhoods as tight regions, one can ensure that the attributes proximate to the neighborhood are proximate to everyone in the neighborhood. Second, the everyday contexts where individuals spend time are also central to neighborhood effects. Much of the time that adolescents spend outside of their home is spent in census tracts other than the one they live in, and these everyday activity space exposures are thought to be highly relevant for child development [22]. Everyday contexts may also operate as the physical embodiment of public services, institutional resources, and local market actors that directly impact residents’ well-being.

Indeed, other public health scholarship further suggests that framing a neighborhood in this way is a relevant approach to thinking about health outcomes. A large body of literature implicates both the social and built environment in health outcomes [11, 23], and by focusing on maximizing homogeneity in residential and everyday exposures, I believe my approach to conceptualizing neighborhoods aligns strongly with this theory. For example, environmental exposures, such as air pollution, are relevant for numerous health outcomes [24], and are specific to specific geographical places. Groups of people who reside near one another and spend their time during the day in similar contexts should be exposed to similar levels of air pollution. Similarly, the social features of a neighborhood, such as cultural norms and social capital, are also strongly linked to health [25], and are likely strongly influenced by everyday mobility patterns. Groups of people who spend their everyday lives in similar social contexts will likely experience similar social exposures.

The ability to analyze everyday contexts has recently expanded with the advent of cell phone mobility data. Cell phone mobility data enables social scientists to analyze the types of non-residential spaces individuals spend time at during their everyday life [26]. In this paper, I introduce a novel approach for examining and identifying spatially cohesive communities that have high levels of within-cluster similarity in terms of activity space exposure. This operationalization consists of clusters of contiguous census block groups with similar everyday mobility patterns. By being physically proximal and interacting with a similar set of everyday spaces, I argue that residents within these clusters experience disproportionality similar residential and activity space exposures. Furthermore, I empirically show that this method is exceptional at building clusters that are highly segregated from one another in terms of race, educational attainment, occupation, age, COVID-19 case timing, and criminal offense timing. Ultimately, I argue that the characteristics of the clusters generated indicate that certain meaningful boundaries by which to spatially delineate communities do tend to exist. While I do not explicitly believe these clusters represent “neighborhoods” or that the identified boundaries correspond with any true neighborhood boundary (nor that such boundaries do exist), I do believe that this method offers a novel approach for delineating regions of cities in a theoretically meaningful manner.

I arrange the rest of the paper as follows. First, I describe the data sources involved in these empirical analyses. Subsequently, I lay out methods and results for a series of four empirical analyses. The first empirical analysis explores the association between demographic similarities and similarity in mobility patterns for pairs of contiguous census block groups. The second empirical analysis introduces the novel clustering procedure and explains how the procedure’s outcome can be effectively viewed as a “test” of whether meaningful, non-gradational clusters of census block groups exist. The third empirical analysis examines the characteristics of these clusters, demonstrating that they are exceptionally segregated in demographic terms. The fourth empirical analysis examines how underlying social processes vary exceptionally between these clusters, as made evident by the division in the timing of COVID-19 case incidence and criminal offending. I conclude by discussing the overall results in the context of the neighborhoods literature and recommending future directions for research.

### Data

Data for this project originates from four sources. First, mobility data is obtained from SafeGraph’s “Monthly patterns” dataset. SafeGraph is a company that obtains the rights to mobile phones’ location data, thus enabling them to track a panel of 40 million mobile devices in the United States as they go about their daily lives. The “Monthly Patterns” dataset consists of tabulated monthly data on the number of visitors to a set of 5 million points of interest in the United States from a set of 220,000 residential census block groups. Each mobile device’s residential census block group is estimated based on the common nighttime location of the mobile device. A visit to a point of interest is estimated by one or more pings within the point of interest’s geographical footprint. I sum the number of visitors from a census block group to a point of interest over the 12 months of 2019 to obtain yearly mobility patterns. One limitation of the SafeGraph “Monthly Patterns” dataset is that it does not include data on visits to residential locations.

The third source for the data is the 2015–2019 American Community Survey 5-year estimates. Data on census block groups’ age distribution, racial composition, occupational makeup, educational attainment, and income distribution are obtained. The fourth source is the 2010 shapefiles for United States census block groups. Spatially contiguous census block groups are identified based on these shapefiles.

The fifth and sixth sources of data applied in this project pertain to the Milwaukee-specific analysis. Specifically, COVID data is obtained from the Wisconsin Department of Health, and crime data is obtained from the Milwaukee Police Department’s Wisconsin Incident-Based Report (WIBR) system. The COVID data contains historical census tract data on positive tests by day. From this data, I estimate the number of new positive tests for 37 weeks in 2020 (all that was available for 2020) for each census tract. The WIBR data contains a list of criminal offenses by census tract, date, and type in Milwaukee. I aggregate this data into two types of offenses: violent and property. I then aggregate the number of incidents of each type by census tract by month for every month in 2019.

## Methods

### Correlation analysis

My first analysis examines what demographic similarities predict similarities in mobility patterns. For all contiguous pairs of census block groups in the U.S., I estimate Pearson correlation coefficients across five variables: age distribution, racial composition, occupational makeup, educational attainment, and income distribution. These correlation coefficients are calculated based on the vectors that characterize the number of census block group residents that fall into specific categories within each type of demographic attribute. As a simple example, if the demographic attribute is Age, there are only three Age categories, and for a pair of census block groups, *i* and *j*, the number of residents of each census block group that fall into the three categories are 4,5, and 6 residents and 7,4, and 5 residents, respectively, the correlation here would be the Pearson correlation between those two vectors of values, or -0.65.

As a dependent variable, I estimate the Pearson correlation coefficient between mobility patterns. For a pair of census block groups, *i* and *j*, this correlation is measured across the number of visits residents of each census block group make to all points of interest in the commuting zone. If the residents of both census block groups tend to allocate visits to the same set of places at similar rates, the correlation coefficient will be positive and large. If the residents of both census block groups tend to visit different points of interest or at different rates, the correlation coefficient may be small or even negative. I choose the Pearson correlation coefficient because it is easily interpretable and since adjacent census block groups are unlikely to have widely different visit distributions, it can accurately capture small differences in visit distributions. I estimate correlation in mobility patterns between adjacent block groups as a product of correlation in demographic characteristics:

$${MOB}_{ij}=\beta_{1}*{AGE}_{ij}+\beta_{2}*{RACE}_{ij}+\beta_{3}*{INC}_{ij}+\beta_{4}*{EDUC}_{ij}+\beta_{5}*{OCC}_{ij}+\nabla_{k}+\varepsilon_{ij}$$

Where *i* and *j* are adjacent census block groups, *MOB*_*ij*_ is the Pearson correlation coefficient between the two census block groups’ mobility patterns, *AGE*_*ij*_ is the Pearson correlation coefficient between the two census block groups’ age demographics, *RACE*_*ij*_ is the Pearson correlation coefficient between the two census block groups’ racial demographics *INC*_*ij*_ is the Pearson correlation coefficient between the two census block groups’ household income distribution, *EDUC*_*ij*_ is the Pearson correlation coefficient between the two census block groups’ educational distribution (among individuals aged 25 and over), *OCC*_*ij*_ is the Pearson correlation coefficient between the two census block groups’ occupational distribution, ∇_*k*_ are commuting-zone fixed effects and *ε*_*ij*_ is an error term with the usual assumed statistical properties.

### Cluster generation

In my next analysis, I explore the meaningfulness of neighborhood boundaries using a novel test. To begin, I will conceive of census block groups in each commuting zone as an undirected network. In these undirected networks, I define the presence of a tie between any two census block groups depending on whether they are spatially contiguous. In the absence of the two census block groups being spatially contiguous, no tie between them exists. Constructing networks in this manner, most commuting zones constitute a single component. That is, a path of some length exists that can connect any two census block groups. Next, let us consider what might happen if we start deleting ties at random. While deleting a single tie might not divide the network into two components, deleting a large number of ties certainly will. Deleting more ties, the number of components the network has will incrementally increase until all census block groups resemble spatial isolates.

Such a procedure is the principal intuition behind this test of whether meaningful boundaries within cities exist. Consider a similar procedure, except instead of deleting ties at random, ties are deleted based on the mobility pattern correlation associated with the tie. Ties are deleted in a predetermined order in this way, with ties with the lowest mobility pattern correlations being deleted first and ties with the highest mobility correlations being deleted last. (The volume of visits that the mobility patterns are based off of is very large. Census block groups average 19,009 visits to POIs in the dataset, and the variation in adjacent census block group’s correlation is quite substantial (IQR: .603: .847), so it is unlikely that the order of correlations is very sensitive to small changes in the data.) If this approach builds a greater number of unique components faster than deleting ties at random, that would suggest that cohesive clusters of census block groups with uniquely patterned everyday mobility habits exist. On the other hand, if the number of unique components is built at the same speed as they would if ties were deleted randomly, that would suggest that census block group mobility patterns simply vary gradationally over space. Ultimately, the principal intuition for this test relies on the notion that while lines can be drawn arbitrarily between neighborhoods, abrupt shifts in mobility patterns that separate clusters of census block groups from one another indicate a more meaningful division in underlying social processes. These divisions, if they do exist, would seem to constitute an ideal set of lines by which to divide areas within cities from one another.

### Nationwide cluster analysis

While the cluster generation process can provide insight into whether the clusters being generated are meaningful, I also need to know when to terminate the procedure and settle on a set of clusters. For exploratory purposes, I will utilize a data-driven approach for identifying the ideal number of clusters. The intuition behind the clustering algorithm is that if there are stark underlying clusters of census block groups in terms of mobility patterns, this clustering algorithm should first delete all of the ties that connect adjacent census block groups between clusters (the ties that should be deleted), before deleting all of the ties that connect adjacent census block groups within clusters (the ties that should not be deleted). As the algorithm runs, I expect to see the number of clusters grow quickly before the clusters are separated, and then after they are fully separated, I expect to see growth in the number of clusters slow down as the tight-knit clusters require more tie deletions in order to be broken apart. While I do not know what optimal clusters do exist, I can infer at what iteration in the algorithm those clusters are likely to exist at based on an inflection point in the rate of growth in the number of clusters. To contextualize the rate of growth in the number of clusters, I compare it to the rate of growth observed if ties were deleted at random. The stopping point is the point at which the number of clusters is maximally different from what would be expected at random, since this point implies that rapid growth in the number of components had occurred before that point and slower growth will happen afterwards.

In either generation procedure, there is only one component when the procedure starts and the maximal possible number of components when the procedure stops. Subsequently, if I settle on the set of clusters generated too early, there will be very few, and thus, the clusters will likely be large and uninformative. Similarly, meaningful clusters may be broken up if I wait too long, and the small clusters or isolates that remain may also be uninformative. Therefore, I settle on the cutoff point as the iteration in the procedure where the number of components in the spatial contiguity network is *maximally larger* than the mean number of components for that iteration in the random deleting procedure. This maximum point is estimated based on 100 samples (limited to 100 due to computational demand) of deleting random ties sequentially. (The clusters derived from this procedure do not necessarily have the absolute properties outlined earlier. Children within the clusters likely do not all attend the same set of schools and workers do not all work at the same workplace. The central properties of the clusters are that they are spatially contiguous and all census block groups within the clusters have relatively similar everyday mobility patterns.) It is important to note that alternative stopping rules can be implemented. In particular, if one desires clusters of a certain size or that there is a specific total number of clusters, one can simply choose a different stopping rule, such as terminating the iterative procedure when the desired number of clusters has been reached. For the purposes of this analysis, however, I will analyze the clusters generated with this data-driven stopping rule.

Using 2000 Commuting Zone definitions, I perform this procedure on each commuting zone in the United States with at least 100 census block groups. To evaluate the meaningfulness of the clusters created, I measure Theil’s entropy index across five groups: age groups, racial/ethnic groups, household income groups, educational attainment groups, and occupational groups based on the clusters. This effectively measures group segregation for each demographic category between clusters. I then compare those entropy indices to ones obtained from random clusters of the same *N*. For each commuting zone, I perform 100 repetitions of the random deletion procedure for clustering.

Income, race, education, occupation, and age demographic data are meaningful and salient demographic categories by which to distinguish sets of census block groups from one another as they follow from a long line of neighborhood research on the focal attributes by which residential segregation persists. Race, in particular, is a dominant facet of how urban areas in the United States are spatially and socially organized [27]. More recent research has highlighted the growing role of income in urban spatial organization [28]. Proximal neighborhoods with vastly different demographic compositions have been shown to be linked with vastly different outcomes for residents [29]. Ultimately, if mobility-generated clusters are exceptionally segregated in demographic terms, this would indicate that these clusters as a construct possess external validity by being correlated with a set of divisions with which past scholars have argued urban subareas should be distinguished from one another.

The clusters generated from this procedure have two properties that have seldom, if ever, been combined in the same neighborhood operationalization. Specifically, the census block groups generated from this procedure are 1) spatially contiguous and 2) have strong similarity in terms of everyday mobility patterns. Most past methods for clustering have either clustered census block groups solely for the sake of spatial contiguity or to maximize network modularity without requiring spatial contiguity, but not both. As a result of the unique properties of the clusters derived from this method, I would further emphasize that the central benefit of this clustering procedure are these properties and the fact that they align so closely with the theoretical properties that a neighborhood operationalization should have. As Diez Roux and Mair write, “researchers should define the spatial context relevant to the health outcome being studied based on theory about the processes involved” [11]. As past literature suggests that residential location and everyday contexts are important and at the heart of the spatial context relevant to many outcomes, health or social, this clustering procedure is uniquely valuable because it so closely aligns with the properties that are relevant to underlying theoretical processes.

As a point of clarification, I would emphasize that the clusters generated from this procedure do not optimally maximize network modularity. Rather, the intuition behind the clustering approach is to jointly provide a test of the presence of meaningful boundaries between sets of census block groups, as well as generate clusters. I view generating census block group clusters that have optimal network modularity and are spatially contiguous as a computationally challenging problem that is beyond the scope of this analysis.

I hypothesize that this clustering algorithm will work less effectively in commuting zones of certain sizes. In the smallest commuting zones, a greater proportion of census block groups tend to be located on the perimeter of the commuting zones—and subsequently have a lower count of spatially adjacent census block groups. These census block groups with fewer spatially contiguous neighbors require fewer cuts to be disconnected—and consequently are disproportionately likely to be disconnected sooner—in spite of the fact that they might not display as distinct of mobility patterns as other spatially contiguous sets of census block groups.

I additionally hypothesize that the clustering algorithm may not work as effectively in the largest commuting zones. The spatial contiguity matrices for census block groups in the largest commuting zones constitute networks with thousands of nodes and tens of thousands of edges. Breaking up such networks into equal-sized components requires an exceptionally greater number of cuts. Spatially contiguity matrices inherently have very high clustering coefficients. In the largest commuting zones (which correspond to the largest networks), a high clustering coefficient induces a high level of redundant ties, which can compound over many layers and make it extremely difficult to break apart equal-sized components. Similar issues have been documented previously in spatial clustering algorithms, where resulting clusters are unreasonably large in very large metropolitan areas [30]. As a result, I hypothesize that the clustering algorithm will also work less effectively on the largest networks—being more likely to result in dissimilarly sized components.

### Milwaukee cluster analysis

I additionally measure Theil’s entropy index across two other attributes: weekly COVID cases and monthly crimes of two types (Violent and Property). Due to data limitations (COVID data is only available at the census tract level for a few jurisdictions nationwide), I perform this analysis for only one geographical area, the city of Milwaukee, Wisconsin. In these analyses, different groups correspond to different weeks of COVID cases and different combinations of crime types and months. Since data originally exists at the census tract scale, I impute the number of cases to the block group level by dividing the census tract totals by the number of block groups that make up each census group. (This is a potential violation of the ecological fallacy. Unfortunately, aggregating mobility data to the census tract scale is problematic given how drastically it would shift the size of geographical units and the scale of the queen contiguity matrix. I would note that the point of this analysis is simply to provide an exploratory assessment that considers non-demographic variables. In order for the ecological fallacy violation to substantially bias the results, that would mean that there would be substantial within-census tract variation in crime and COVID-19 and that variation would need to be inversely correlated with the boundaries the clustering identifies. Such variation seems unlikely.) For example, if a census tract comprises three block groups, each block group has one-third of the tract’s COVID cases imputed for each specific week. To evaluate the performance of the clusters in segregating across these two phenomena, I compare the results to 1000 repetitions of the random deletion procedure.

COVID-19 incidence data is a meaningful type of data by which to attempt to distinguish sets of census block groups from one another in terms of segregated social processes. Past research on COVID-19 incidence has demonstrated that epidemiological simulations of infection accurately match real-world trends and that differences in systems of behavior and interaction drive inter-neighborhood disparities [26, 31–33]. Other research has put forth that differences in collective efficacy and social disorder in mobility patterns can drive disparities in case incidence [34]. Broadly, sets of census block groups that experience different incidences at different points in time are likely socially disconnected and experiencing vastly different underlying social processes.

Analyzing criminal offense timing data is also valuable for understanding the disparities in social processes between neighborhoods. Criminologists posit that a variety of underlying social processes lead to the concentration and diffusion of violence and crime. Social Network Analyses of co-offending networks indicate that gun violence diffuses across social networks [35]. Violence has also been shown to have short-term linkages to environmental exposures, behaviors, and cultural practices, inducing synchrony in violence [36]. Recent research has even highlighted how neighborhood mobility patterns can predict the short-term diffusion of violent crime [37]. Sets of census block groups that experience different types of offending at different points in time are likely socially disconnected and experiencing vastly different underlying social processes.

## Results

### Correlation models

Table 1 presents a correlation matrix for the variables in Table 2—which presents the results of the mobility pattern correlation models. To reiterate, these models predict the correlation in mobility patterns between pairs of census block groups for all contiguous pairs in the United States. Model one includes five predictors: the pair’s correlation in terms of income, race, education, occupation, and age. As expected, most of the five have a strong positive association with mobility correlation. Race has the greatest effect; a pair of census block groups with perfectly positively correlated racial compositions would be expected to have .44 more correlation in mobility patterns than a pair of census block groups with perfectly negatively correlated racial compositions. In descending order of magnitude, education, occupation, and age correlations all also have a highly significant positive association with mobility correlation. Income correlation, surprisingly, has a slight, negative relationship with mobility correlation. However, I attribute this negative effect to overfitting since income is closely related to education and occupation.

**Table 1:** Correlation matrix.

	Mobility Correlation	Income Correlation	Race/Ethnicity Correlation	Educational Correlation	Occupational Correlation	Age Correlation
Mobility Correlation	1	0.032	0.206	0.2	0.133	0.092
Income Correlation	0.032	1	0.111	0.144	0.199	0.122
Race/Ethnicity Correlation	0.206	0.111	1	0.362	0.251	0.142
Educational Correlation	0.2	0.144	0.362	1	0.167	0.093
Occupational Correlation	0.133	0.199	0.251	0.167	1	0.196
Age Correlation	0.092	0.122	0.142	0.093	0.196	1

**Table 2:** OLS models predicting mobility correlations with demographic correlations.

	Model 1	Model 2	Model 3	Model 4
Income Correlation	-0.008 ***	0.001		
	(0.001)	(0.003)		
Race/Ethnicity Correlation	0.220 ***	0.192 ***	0.221 ***	0.193 ***
	(0.003)	(0.015)	(0.003)	(0.015)
Educational Correlation	0.095 ***	0.077 ***	0.094 ***	0.078 ***
	(0.001)	(0.006)	(0.001)	(0.005)
Occupational Correlation	0.044 ***	0.073 ***	0.042 ***	0.075 ***
	(0.001)	(0.010)	(0.001)	(0.010)
Age Correlation	0.023 ***	0.029 ***	0.023 ***	0.030 ***
	(0.001)	(0.003)	(0.001)	(0.003)
CZ Fixed Effects		X		X
N	478884	478884	479689	479689
AIC	-331434.124	-384631.198	-330037.788	-383480.180
BIC	-331367.649	-379856.057	-329982.383	-378715.396
Adj. R2	0.068	0.167	0.069	0.168

*** p < 0.001

** p < 0.01

* p < 0.05.

Model 2 adds in Commuting zone fixed effects. These fixed effects control for unmeasured variation in the tendency for proximal census block groups to have especially similar or different mobility patterns. While this model slightly attenuates the effect of race and educational correlations, the size of the coefficients for occupational and age correlations actually increases. In addition, the effect of income correlation is attenuated beyond statistical significance. Models 3 and 4 replicate models 1 and 2 without the income correlation coefficient, revealing similar results.

### Cluster generation

**Fig 1:**
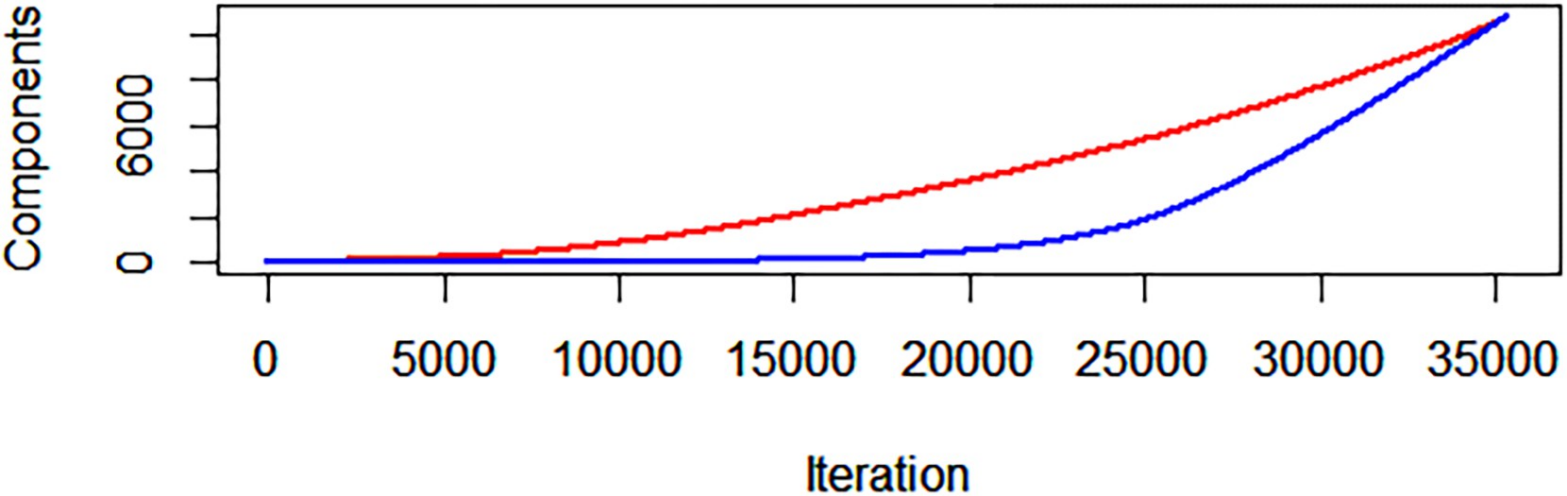
New York City cluster generation.

The cluster generation procedure reveals that components are created much more quickly by deleting ties in ascending order of mobility correlation compared to at random. Fig 1 displays the number of components by iteration for the New York City commuting zone, the largest in the United States. The red line indicates the number of components at each iteration of the mobility-clustering procedure. The blue line indicates the mean number of components based on 100 repetitions of a random-clustering procedure. The difference in component generation speed is stark. In the middle range of the figure, the mobility-clustering procedure had more than 3,000 more components than the random-clustering procedure. This New York City-specific result aligns with the results observed for all commuting zones in the United States. For every one of the 349 commuting zones in the United States with at least 100 census block groups, I observe that this procedure divides each spatial contiguity network into components far more quickly than what would be predicted to occur at random. At the halfway mark of iterations, there was a median of 4.985 times as many components generated than at random, with a minimum of 2.473 and a maximum of 12.054. These results indicate that substantial clustering does exist in terms of mobility patterns. Patterns of movement do not flow gradationally across census block groups; instead, sharper lines exist by which clusters of census block groups can be easily divided.

Nationwide, the median commuting zone has an average of 11.6 census block groups in a cluster. The scale of a cluster is, thus, somewhat larger than how a conventional “neighborhood” might be operationalized. Cutchin and colleagues [38] assert that, in the United States, “the most frequent operationalization of the neighborhood in the United States is the census tract.”. Census tracts, on average, contain 2.98 census block groups. This suggests that the typical cluster size is substantially larger than the typical census tract size and, subsequently, what might be considered to be the typical neighborhood scale. The size of clusters also varies somewhat between commuting zones, with the interquartile range extending from 8.7 to 16.2.

### Nationwide cluster analysis

**Fig 2:**
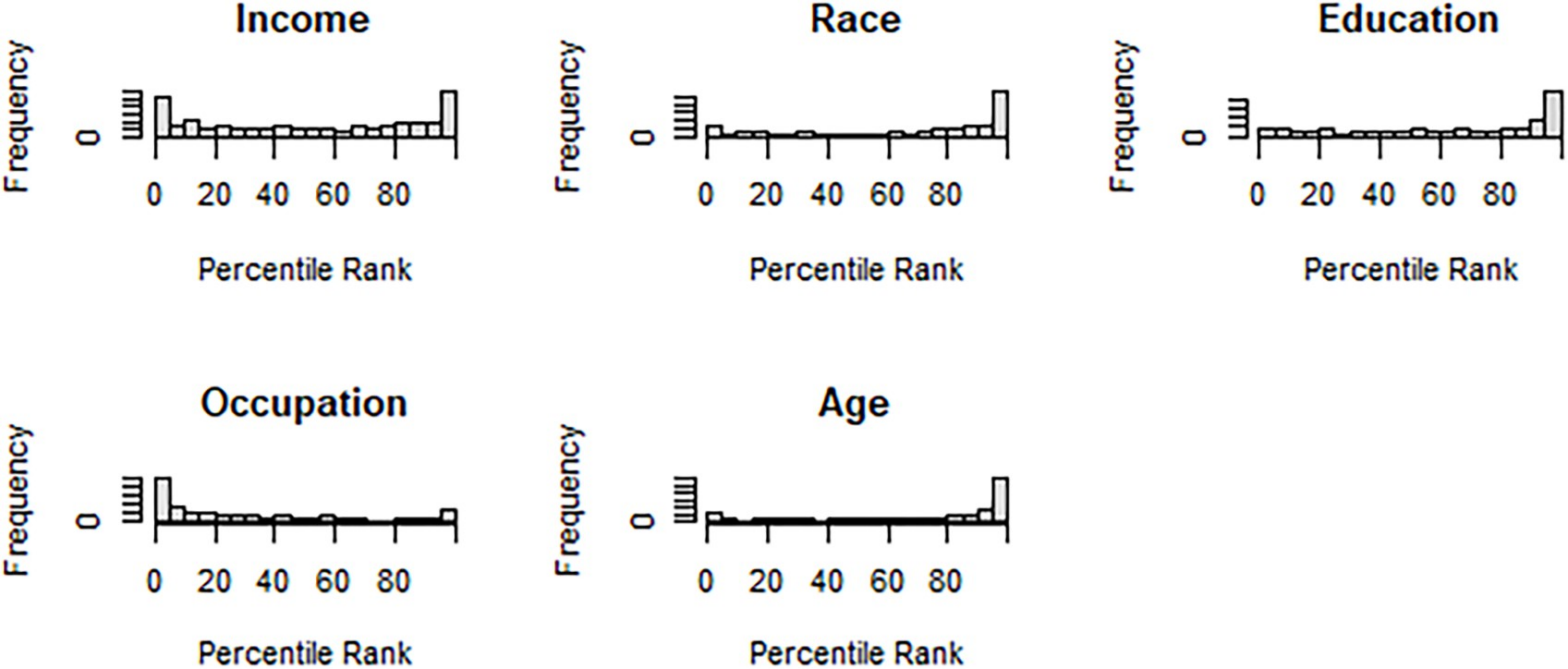
Cluster segregation histograms across commuting zones.

Table 3 presents the cluster segregation analysis results, and Fig 2 presents these results graphically in a histogram format. For each of the 349 commuting zones, each row of Table 3 presents summary statistics on the rank of the mobility clustering procedure compared to 100 randomly generated clusters. A value of 0 indicates that the mobility-generated clusters were less segregated than all 100 randomly generated clusters. A value of 100, oppositely, indicates that the mobility-generated clusters were more segregated than all 100 of the randomly generated clusters.

**Table 3:** Segregation rank of mobility-generated clusters compared to random clusters.

Segregation Variable	Min.	1st Qu.	Median	3rd Qu.	Max.
Income	0	15	52	88	100
Race	0	40	83	98	100
Education	0	38	76	96	100
Occupation	0	4	19	55	100
Age	0	48	86	99	100
All	0	33	66	90	100
Race+Education+Age	0	50	86	99	100

*Based on 349 Commuting Zones with a minimum of 100 census block groups.

The income segregation analysis results reveal that the mobility-generated clusters’ performance is on par with the average randomly generated cluster, but the results are widely distributed. In some commuting zones, the mobility-generated clusters are exceptionally segregated in income, while others are exceptionally integrated. The results of the race segregation analysis reveal more striking evidence that the mobility-generated clusters are especially segregated in terms of race. The mobility-generated clusters in the median commuting zone are more racially segregated than 83% of randomly generated clusters. In more than one-quarter of commuting zones, the mobility-generated clusters are more racially segregated than at least 98% of randomly generated clusters. The results for educational segregation are similar.

The results for occupational composition are shockingly skewed towards integration. Mobility-generated clusters are disproportionately more integrated in terms of occupation than randomly generated clusters. In over one-quarter of commuting zones, the mobility-generated clusters are more segregated than no more than 4% of randomly generated clusters. Somewhat surprisingly, age-based segregation is captured by the mobility-generated clusters better than segregation in both race and educational attainment. While a large body of literature has examined urban segregation in race and class, less work has looked at age. These results suggest that age is an understudied dimension of segregation.

Table 4 explores heterogeneity in segregation fit by commuting zone size. As hypothesized earlier, in the smallest commuting zones, mobility patterns seem less likely to structure divisions between segregated neighborhoods because they are less likely to harbor macrosegregation and less likely to have the capacity for fragmented mobility patterns to emerge. The largest commuting zones, on the other hand, also seem less likely to have mobility-generated clusters capture macrosegregation because the generation procedure seems not as endearing to large spatial contiguity networks, and oddly divided components seem more likely to emerge.

**Table 4:** Segregation rank by commuting zone size.

Size	Variable	Min.	1st Qu.	Median	3rd Qu.	Max.
Small	Race	0	38	81	97	100
Small	Education	0	43	74.5	95	100
Small	Age	0	44	83	98	100
Medium	Race	0	73	96.5	100	100
Medium	Education	0	37.25	89.5	100	100
Medium	Age	0	61.5	96	100	100
Large	Race	0	0.5	20	98.5	100
Large	Education	0	3.5	34	100	100
Large	Age	0	49	99	100	100

N is 256, 74, and 19 for Small, Medium, and Large commuting zones, respectively.

Table 4 presents results exploring this. My hypotheses generally align with these findings. For the medium-sized commuting zones, mobility-generated clusters are exceptionally segregated in terms of race, education, and age, as indicated by the Median and 75^th^ percentile ranking. For small commuting zones, mobility-generated clusters do appear very segregated in terms of race, education, and age, but less so compared to medium-sized commuting zones. For large commuting zones, mobility-generated clusters do appear exceptionally segregated in terms of age, but less so in terms of race and educational attainment. While going into detail with how the clustering algorithm performs for specific commuting zones is beyond the scope of this paper, it is worth noting that for Chicago—a city that has been studied substantially in terms of racial segregation and the spatial isolation of non-White racial groups—the clustering method performs very poorly. In Chicago, the clustering procedure produces clusters that are less segregated in terms of income, race, or education than all 100 of the randomly generated comparison sets of clusters. (Part of the reason that the clustering procedure may not work well in Chicago is because the commuting zone is very large, and subsequently the algorithm fails to produce similarly-sized clusters. The majority of census block groups actually end up as just part of one cluster).

Ultimately, these results suggest external validity to the mobility-generated clusters. These clusters are disproportionally associated with demographic clustering. In many cases, they appear to constitute an exceptional approach by which to cluster proximal census block groups in terms of race, educational attainment, and age composition. The consistent findings across many different commuting zones suggest this clustering procedure is robust to different contexts and also provides suggestive evidence of the approach’s robustness to error in the measurement of mobility patterns (assuming this error is randomly distributed across commuting zones).

As an alternative clustering procedure, I additionally cluster census block groups (by commuting zone) on mobility patterns using k-means, where *k* is the analogous number of clusters generated using the original method here. Table 5 presents the proportion of commuting zones for which there is higher segregation using the original method compared to the aspatial k-means approach. As the table shows, the original approach here performs significantly better in terms of producing segregated clusters in terms of all categories, except for occupation, where the difference is insignificant. These results suggest that the unique combination of clustering on both space and mobility patterns, rather than just mobility patterns, performs better in terms of identifying homogenous clusters of census block groups.

**Table 5:** Percentage of commuting zones where novel approach outperformed k-means.

	Better	Lower Bound	Upper Bound
Income	61.6%	56.4%	66.8%
Race	71.6%	66.8%	76.2%
Education	77.9%	73.6%	82.2%
Occupation	49.3%	44.1%	54.4%
Age	73.6%	69.1%	78.2%
All	78.2%	73.9%	82.5%
Race+Education+Age	83.7%	79.7%	87.4%

Lower and Upper Bounds refer to 95% Confidence Intervals

Table 6 presents statistics on the distribution of the size of clusters in the main clustering approach. Similar to other spatial clustering methods [30], unreasonably large clusters appear to be prominent. These exceptionally large clusters are especially common in large commuting zones. Furthermore, the table indicates that the modal cluster appears to be an isolate—composed of a single census block group. Despite these issues, the comparison results still indicate relatively high modularity in the clusters produced, suggesting that the general clustering approach is highly promising. Modified methods that produce more evenly distributed clusters may yield even better results. Potential solutions to address unevenly distributed cluster sizes are presented in the discussion.

**Table 6:** Distribution of cluster sizes by commuting zone size.

All Commuting Zones		
Size	Percentage of Clusters	Percentage of Census Block Groups
1	72.60%	4.30%
2 to 10	17.50%	3.90%
11 to 100	7.60%	15.50%
101 to 1000	2%	35%
1001 to 5000	0.30%	30.30%
5001 to 10087	0%	11.10%
Small Commuting Zones		
Size	Percentage of Clusters	Percentage of Census Block Groups
1	60.40%	5.60%
2 to 10	23.60%	9.10%
11 to 100	13.60%	44%
101 to 1000	2.40%	41.30%
1001 to 5000	0%	0%
5001 to 10087	0%	0%
Medium Commuting Zones		
Size	Percentage of Clusters	Percentage of Census Block Groups
1	80.60%	4.90%
2 to 10	13.80%	2.90%
11 to 100	3.20%	6.90%
101 to 1000	2%	56.40%
1001 to 5000	0.40%	29%
5001 to 10087	0%	0%
Large Commuting Zones		
Size	Percentage of Clusters	Percentage of Census Block Groups
1	86.80%	2.50%
2 to 10	9.40%	0.90%
11 to 100	1.90%	1.50%
101 to 1000	0.90%	8.70%
1001 to 5000	0.90%	55.50%
5001 to 10087	0.10%	30.90%

### Milwaukee specific analysis

The results of the Milwaukee analysis confirm the extent to which criminal offending and infectious disease incidence are especially segregated between mobility-generated clusters. Of the 1,000 randomly generated sets of clusters, 993 had lower segregation in terms of criminal offending than the mobility-generated clusters. Similarly, 959 had lower segregation in terms of COVID incidence than the mobility-generated clusters. Only 13 randomly generated cluster sets had a higher combined rank across these two phenomena than the mobility-generated clusters. This Milwaukee-specific analysis provides additional evidence that mobility-based clusters capture meaningful differences in neighborhood social phenomena and that the procedure produces exceptionally segregated clusters across these important attributes.

## Discussion

Ultimately, the results of these analyses suggest four key findings. First, divisions between census block groups in mobility patterns correlate strongly with divisions in race, educational attainment, occupational composition, and age composition. Demographic divisions and physical boundaries are key to understanding urban segregation, and thus, this finding suggests that segregation in mobility patterns reflects residential segregation. Second, these divisions between census block groups in mobility patterns can entirely separate clusters of census block groups from one another with only a small number of edge deletions. This finding was made evident by my novel cluster generation procedure. The results of the procedure strongly indicate that some boundaries are indeed more meaningful than others.

Third, the clusters generated by dividing census block groups in terms of mobility patterns produced exceptionally segregated clusters in terms of race, educational attainment, and age. This finding further suggests that operationalizing regions of cities as areas with homogenous mobility patterns is not only theoretically informed, but also appears to reflect meaningful demographic segregation. Fourth, the clusters generated by dividing census block groups in terms of mobility patterns produced exceptionally segregated clusters in terms of COVID-19 incidence timing and criminal offending type and timing. This finding, in particular, confirms that the clusters this procedure generates align with divisions in key underlying social processes that theory suggests should distinguish neighborhoods from one another.

Ultimately, these findings broadly contribute to a critical dialogue surrounding what neighborhoods are, both in conceptual and operational terms. While a large body of sociology research has glossed over neighborhood operationalization and simply relied upon arbitrary definitions by the Census Bureau, other recent research has encouraged scholars to be more thoughtful and use egocentric neighborhoods with overlapping boundaries, implicitly eliminating rigid boundaries altogether [6]. While egocentric neighborhoods address several important problems with past operationalizations of neighborhoods, their fluidity makes them a weaker tool for empirical analyses. Rather, as I show here, there do appear to be more meaningful boundaries with which to divide up geographical areas for analytic purposes. While I do not believe these clusters represent objective “neighborhoods,” these clusters may be a useful unit of geography with which to study social processes.

Broadly, while the clusters generated by this novel procedure demonstrated a fairly effective method with which to partition census block groups in terms of demographics, crime, and infectious disease incidence, I would again belabor the point that the central utility of this clustering procedure as a method for operationalizing geographical units of analysis lies in its unique theoretical properties. Specifically, this novel clustering procedure produces neighborhood clusters that are both spatially contiguous and are made up of residents who have similar everyday mobility patterns. These two properties are theoretically motivated based on the attributes of neighborhoods that past literature has implicated as important for understanding neighborhood-level effects on individual outcomes.

One limitation of this clustering procedure is the tendency for the algorithm to produce unreasonably large clusters in some situations. I suggest two simple solutions to address the issue of unreasonably large clusters. First, one can artificially reduce the set of census block groups that the algorithm clusters over. For example, in my application, I clustered across all census block groups in each commuting zone. An alternative to this would be to cluster across all census block groups in each county. Since counties are smaller than commuting zones, the largest components will be substantially smaller if clusters between different counties are not permitted.

The second solution for reducing unreasonably large clusters is simply to run the algorithm longer. The size of the largest component monotonically decreases as more iterations of the algorithm run. The algorithm can theoretically run until all census block groups are part of separate isolate clusters. The stopping rule I recommended is principled for identifying latent clusters, but may not always work well or be suited for all needs. An alternative stopping rule may involve choosing a pre-determined cluster size limit and then terminating the algorithm when all clusters are below that limit. Results for this paper employing either of these solutions are available upon request to the author.

In addition to the two aforementioned solutions, the algorithm could also be modified to artificially delete additional edges when two sets of census block groups are nearly disconnected. There are likely many instances in the algorithm where two sets of census block groups remain a single component despite having had a substantial number of internal ties deleted and being nearly disconnected. Future modifications of this algorithm may attempt to search for these situations after each iteration and delete edges (sooner than they would otherwise be) in order to separate nearly disconnected sets of census block groups fully. The exact implementation and effectiveness of this approach remain uncertain, as it is not clear how best to identify and handle these nearly disconnected sets of census block groups.

Broadly, this research has substantial implications for how neighborhoods are conceptualized and operationalized in the study of health. The properties of the census block group clusters align closely with theories regarding the relationship between neighborhoods and health [11, 25]. It may be useful for these clusters to be applied to the study of health outcomes as a geographical unit of analysis. These clusters may help inform in what cohesive neighborhoods disparities in health exist and how interventions can be applied to alleviate them. The bimodal nature of the underlying mobility networks that characterize these clusters also means that public health interventions may be leveraged in terms of both residential locations and everyday contexts, potentially increasing the effectiveness of such interventions. The clustering approach outlined here may also be useful for measuring and studying resiliency in mobility networks. Analyses of the clusters identified here in terms of the network properties that are beneficial for resilience could complement other recent research [39]. This approach may also serve as a highly useful tool with which to measure and study segregation in mobility networks more broadly.

The results of these analyses do beg several questions for future research. First, this work uncovered race, education, and age as focal demographic attributes separating neighborhoods from one another. Future work exploring the importance of age distribution in urban spatial segregation will be imperative to understanding neighborhood structure. Broadly, the cluster generation procedure results suggest that substantial clustering exists in mobility patterns, and future research should investigate the underlying causes of this clustering and how exactly it is related to other forms of urban segregation. Future research could also specifically investigate the role that educational access and school attendance play in clusters. Past research has documented housing choices for educational access purposes to be a chief driver of residential segregation in terms of income [40]. It is also possible that school attendance patterns may play a role in age segregation between clusters. Future research should better disentangle these mechanisms.

The cluster segregation analysis results additionally reveal that the mobility-generated clusters’ performance does vary across commuting zones, opening the door for scholars to investigate how mobility patterns influence segregation differently across different regions. Furthermore, it is important to investigate how these clusters evolve over time and whether different policy interventions might be effective in influencing the level of integration or segregation within these clusters. Statistically, the development of similar procedures for generating clusters that optimize neighborhood modularity may be a substantial improvement over the current method as well. Broadly, future research that relies on mobility patterns aggregated to smaller units of geography, or individual-level mobility data, may be useful in an application of this data to create cluster aggregations that more closely align with the typical “neighborhood” scale. It will also be beneficial for future research to consider the degree to which such clustering procedures are sensitive to error in the measurement of visits or random visits. It would additionally be useful for future scholarship to investigate the sensitivity of these results to census block groups being placed in slightly different clusters.

Overall, the findings of this study highlight how mobility patterns can be a valuable tool in identifying relevant geographical units for the study of social life. As recent research has encouraged operationalization shifts away from neighborhood boundaries, I distinctly encourage *more* research to utilize and investigate neighborhood boundaries. Boundaries are crucial to understanding neighborhood division and inequality, which is ultimately what is at the heart of neighborhood effects research. As novel datasets with which to study neighborhoods become increasingly available, tools must concurrently be developed to take advantage of such data. The approach outlined in this paper may serve as a useful starting point for the development of novel methods to studying neighborhoods jointly in terms of residential and mobility characteristics.
